# Is the digital rectal exam any good as a prostate cancer screening test?

**DOI:** 10.3399/bjgp24X736677

**Published:** 2024-03-01

**Authors:** Mike Kirby, Samuel WD Merriel, Oluwabunmi Olajide, Alexander Norman, Nikhil Vasdev, Vishwanath Hanchanale, Michelle Cain, Malcolm Wilkinson, Harley Stephens, Deborah Victor, William Kinnaird, Alison Tree, Amarnath Challapalli, Amy Rylance, (Prostate Cancer UK Clinical Advisory Group)

**Affiliations:** *Trends in Urology and Men’s Health*; president elect, British Society of Sexual Medicine.; Centre for Primary Care and Health Services Research, University of Manchester, Manchester.; Rush Green Medical Centre, Romford.; Cobham Health Centre, Cobham; co-medical director, Surrey and Sussex Cancer Alliance.; Lister Hospital, Stevenage; professor and chair of robotic surgery, University of Hertfordshire.; Liverpool University Hospitals NHS Foundation Trust, Liverpool.; Clatterbridge Cancer Centre NHS Foundation Trust, Liverpool.; Macmillan urology consultant radiographer, Western Health and Social Care Trust, Londonderry.; SABR and IGRT specialist therapy radiographer, University Hospitals Bristol and Weston NHS Foundation Trust, Bristol.; Royal Cornwall Hospitals NHS Trust, Truro.; University College London Hospitals NHS Foundation Trust, London.; The Royal Marsden NHS Foundation Trust and the Institute of Cancer Research, London.; University Hospitals Bristol and Weston NHS Foundation Trust, Bristol.; Prostate Cancer UK.

There is no shortage of references in popular culture to the prostate examination, with many a laugh built on the punchline of the finger up the bum. Interestingly, while cervical, breast, or bowel screening share barriers to uptake around the intimacy of the examination, ‘ick-factor’, or cultural taboos, they have never become comedy tropes — reflecting the uniquely emasculating perception of the rectal examination.

## So, is the rectal examination a good test for prostate cancer?

Short answer, no. As the emphatically titled paper, ‘Digital rectal examination is not a useful screening test for prostate cancer’ concludes: *‘The performance of stand-alone DRE to screen for prostate cancer is poor … Furthermore, DRE does not improve the detection of PSA-screen–detected prostate cancer.’*^1^

The National Institute for Health and Care Excellence (NICE) diagnostic guidelines for prostate cancer changed in 2019 to advise a multi-parametric magnetic resonance imaging (mpMRI) scan as the first investigation following a referral with suspicion of prostate cancer, which should be used to inform decision making on whether to perform a biopsy.^2^ The PROMIS study that informed this guideline change showed 27% of men can avoid a biopsy following an MRI.^3^ The negative predictive value of mpMRI is 90%^4^ and NICE NG131 sets out that men discharged based on a negative MRI should have follow-up prostate-specific antigen (PSA) testing as a further safety net to reduce the risk of missing significant cancer.^2^

**Figure 1.**
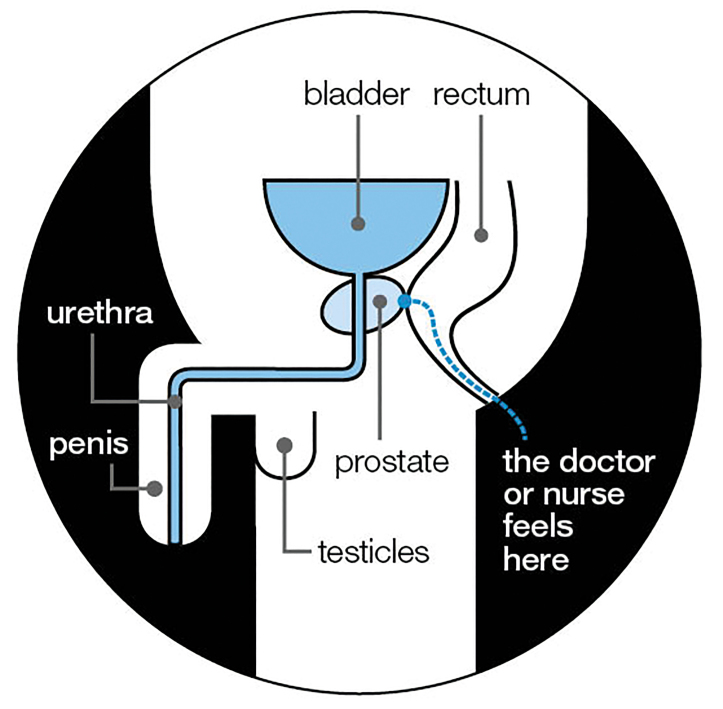
The digital rectal examination. Source: © Prostate Cancer UK. Used with permission. Figure provided by Prostate Cancer UK, January 2024.

Digital rectal examination (DRE) only allows for the clinician to feel the back wall of the prostate gland (Figure 1), so any abnormalities located in the middle or front part of the gland cannot be felt — although these would be visible in an mpMRI. Historically, the DRE may have been a useful way of distinguishing which men may have had a raised PSA caused by benign factors, but in the UK *‘where MRI services are available, the need to perform a DRE routinely should be questioned …’*.^5^

## When is the DRE useful?

Many early-stage prostate cancer cases do not have symptoms.^6^ If a patient does present with lower-urinary symptoms then a DRE can give useful indications of benign prostate conditions, such as benign prostatic enlargement (known as BPE or BPH) — the prostate will feel smooth and significantly larger than a walnut — or prostatitis where the prostate can feel boggy and tender.

In addition, some men with prostate cancer do not produce PSA — around 2 in 100 men with a normal PSA may have an aggressive form of prostate cancer. In these cases, a DRE may detect cancers that the PSA test alone would miss — an abnormal DRE is sufficient grounds to refer for suspected cancer without a raised PSA. An abnormal DRE in symptomatic patients has a positive predictive value (PPV) for prostate cancer well above the current NICE threshold for urgent referral.^7^

There is limited evidence of DRE being an effective screening tool in asymptomatic men with low PSAs.^8^ But it can be an important reassurance for men (or their clinicians) who are particularly concerned about their risk of prostate cancer - for example, because a close relative has died from prostate cancer.

## When is the DRE unhelpful?

Prostate Cancer UK surveyed over 2000 men and 60% of them were concerned about having a rectal exam (personal communication; online survey conducted by OnePoll in April 2023). Of those, 37% would not speak to a GP about prostate worries because of the DRE. The fear of the DRE can be a significant factor in men not talking about prostate cancer. And that deterrent may be larger in the men at higher risk of a late diagnosis — Black men report significantly more cultural stigma around the rectal examination, but face double the risk of dying from prostate cancer.^9^

While a degree of anxiety around the rectal examination is very common, patient insight suggests it can be particularly triggering for men who have experienced sexual abuse either as a child or an adult. Data from the Office for National Statistics report that an estimated 5.7% of men (1.36 million) have experienced sexual assault since the age of 16 and that over 700 000 adult men have experienced sexual abuse before the age of 16.^10^^–^^12^

## What is the future of screening for prostate cancer?

Diagnosing prostate cancer is far safer and more accurate since the introduction of MRI and changes to biopsy technique.^3^ As a result, Prostate Cancer UK strongly recommends that men at highest risk talk to their GP about the PSA blood test. However, there is not yet sufficient evidence to screen all men. Prostate Cancer UK is launching a £42 million research programme — the TRANSFORM trial — to provide the gold-plated evidence required by the National Screening Committee.^13^ It will test multiple screening methods, including MRI scans, to detect prostate cancer. But it will be many years before its results can change practice — and in the meantime GPs will still need to use their clinical skills and the available tests to diagnose prostate cancers earlier and reduce the 12 000 annual deaths from prostate cancer.^14^

## So, to DRE or not to DRE?

The most useful question to ask is, ‘Would it change your clinical decision making?’ If a man has a raised PSA, then the NICE guidance is to refer them on to a suspected cancer pathway. Doing a DRE does not add to your decision making and an MRI will far more accurately indicate whether there are likely benign causes of the PSA being elevated and the man can be discharged without a biopsy.

If his PSA comes back normal, but your clinical suspicions are raised — because of risk factors such as Black ethnicity or family history, or symptoms that might suggest more advanced disease such as lower back pain — then a DRE might detect a suspicious malignancy that could prompt a referral. But the DRE need not be a routine screening test for prostate cancer.
